# Protein S Deficiency: A Case Report

**DOI:** 10.7759/cureus.46864

**Published:** 2023-10-11

**Authors:** Shirley M Gandhi, Pruthvi Patel, James R Conner

**Affiliations:** 1 Internal Medicine-Pediatrics, Alabama College of Osteopathic Medicine, Dothan, USA; 2 Internal Medicine, Alabama College of Osteopathic Medicine, Dothan, USA; 3 Surgery, Helen Keller Hospital, Sheffield, USA

**Keywords:** routine screening, hypercoagulable state, bleeding risk, anti-coagulation, hematological

## Abstract

Protein S is a glycoprotein created by the body that aids in the prevention of a hypercoagulable state. Protein S-deficient patients are placed on anticoagulant regimens, as there is no current definitive cure. Failure to bring balance to the hematological system in these patients will lead to complications such as widespread clot formation and pulmonary embolisms.

Here, we present a 74-year-old female who was admitted to the ICU after collapsing. She presented with respiratory failure, urinary tract infection (UTI), and pneumonia. Magnetic resonance imaging (MRI) scans depicted a thrombus in the distal right transverse sinus and sigmoid sinus. Her hematologic workup showed normal levels of homocysteine, fibrinogen, and protein C levels but protein S levels were reduced to 24%. This case displays the intricate presentation of a rare hematological disease as well as the importance of routine follow-up to maintain patient health.

## Introduction

Advancements in pharmaceutics have led to the development of medications that help blood clot during times of unwanted bleeding and degrade clots to prevent a hypercoagulable state. In addition to these classes of drugs, the human body has its own natural glycoproteins that serve to maintain hematological homeostasis [1]. When faced with tissue trauma and bleeding, the coagulation cascade is triggered, releasing clotting factors that ultimately create fibrin cross-links to prevent further blood loss [2]. This active coagulation process must be countered by anticoagulant proteins to avoid a state of hypercoagulability. This task is enforced by protein C and protein S, which interact with glycoproteins to inactivate clotting factors Va and VIIIa [2].

Deficiencies in protein S can be acquired due to nephrotic syndrome, hepatic disease, disseminated intravascular coagulation (DIC), or vitamin K deficiency [3]. More rarely, protein S deficiency can be passed along generations as an autosomal dominant trait with variable penetrance [3]. The congenital form arises due to a mutation in the PROS1 gene, yielding a premature stop codon and a naive protein S glycoprotein [4]. Heterozygous patients present with a mild form of the disease, with 50% experiencing venous thromboembolic events (VTE) while the other 50% do not get VTEs. On the other hand, homozygous patients have a severe form with an increased risk of VTEs [5].

The prevalence of protein S deficiency is not widely known but is estimated to be between 0.03% and 0.13% in healthy individuals [6]. It has not been determined yet if protein S deficiency has a predilection for either gender, however, it seems that clinical manifestations of VTEs are more likely to present in women due to risk factors such as oral contraceptive pill (OCP) usage, pregnancy, and hormone replacement therapy [3].

## Case presentation

A 74-year-old female was admitted to the medical intensive care unit for seizure/encephalopathy with acute respiratory failure requiring intubation. She was simultaneously combating a urinary tract infection for which she was treated with ceftriaxone. In addition, sputum culture analysis presented the growth of Streptococcus anginosus as the cause of her pneumonia. She had wide QRS ventricular tachycardia, left ventricular ejection fraction (LVEF) of 40%-45% with mild global hypokinesis, and moderate right ventricle dilation with reduced function. Dermatologic stress testing presented normal perfusion and no ischemia but decreased left ventricular systolic function to 49%.

**Figure 1:**
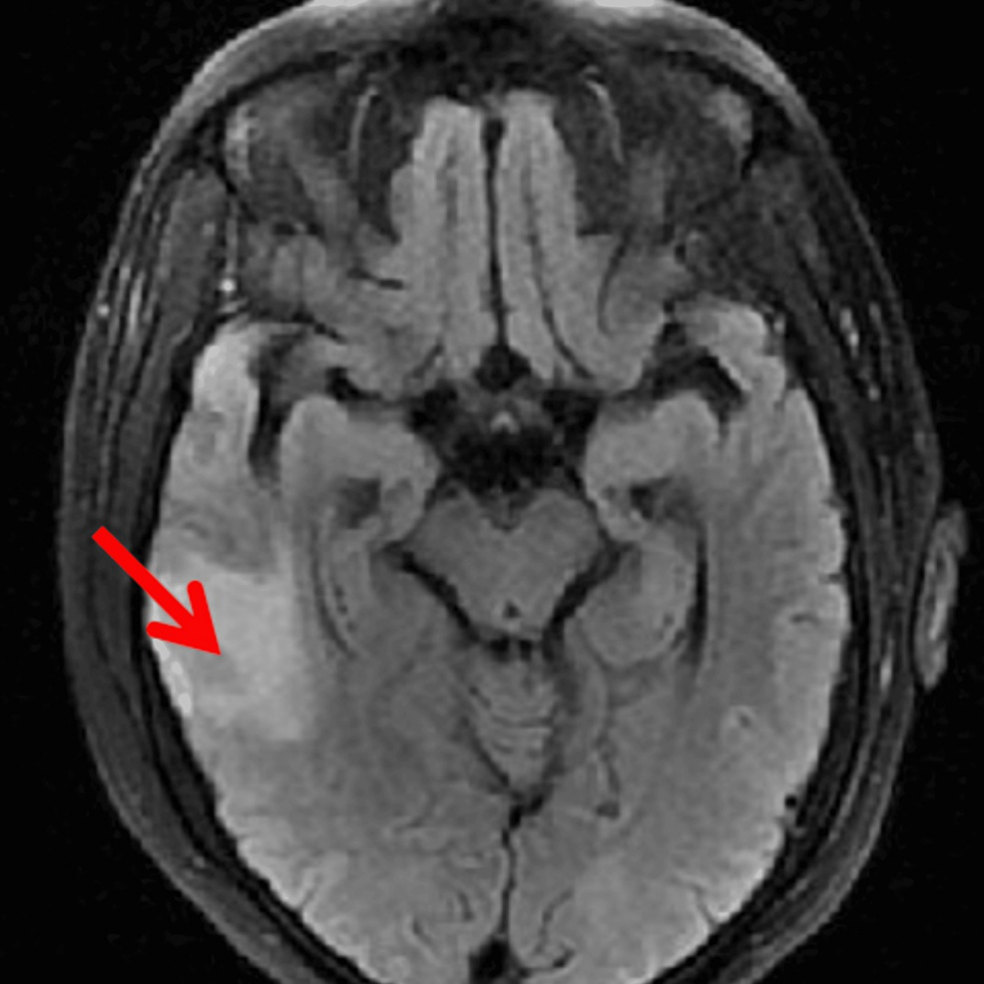
MRI showing cerebral infarction in the right temporal lobe (red arrow)

**Figure 2:**
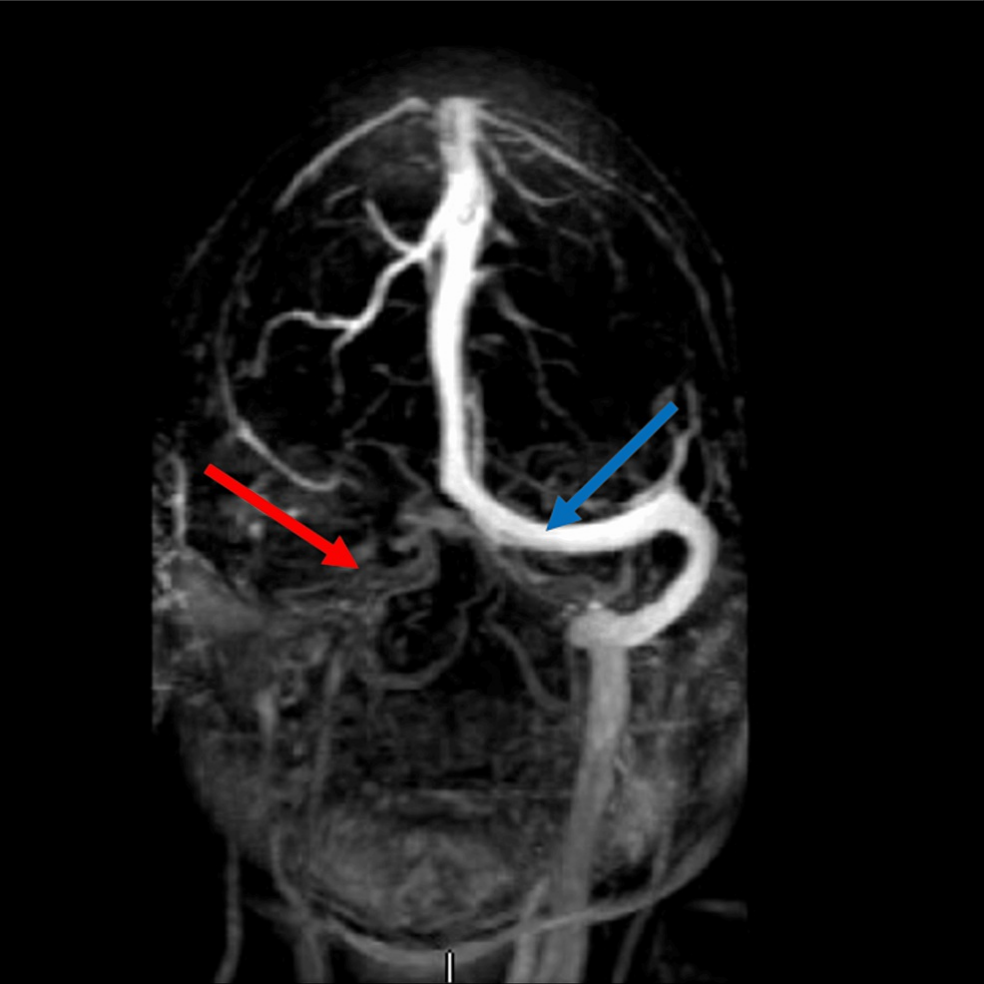
MRV showing absent flow in the distal right transverse and sigmoid sinus (red arrow) in comparison to the left (blue arrow) MRV: magnetic resonance venography

A computed tomography (CT) scan revealed an infarct in the right temporal lobe followed by magnetic resonance imaging (MRI) with and without contrast showcasing diffuse enlargement and edema of the right temporal lobe without a mass (Figure 1). Prior MRI with and without contrast was conducted due to the patient's chief complaint of “memory problems” and was deemed to have nonspecific findings. During her hospitalization, she had unremarkable complete blood count (CBC), comprehensive metabolic panel (CMP), and spinal tap. She underwent a follow-up MRI that showed worsened edema and a suspected thrombus in the distal right transverse sinus and sigmoid sinus. Magnetic resonance venography (MRV) of the brain further confirmed this presentation (Figure 2).

The patient had no prior history of arterial or venous thrombosis. During her stay in the hospital, there was no sinus infection, signs of dehydration, or intake of hormone replacement therapy. There was a family history of blood clots. Hematologic workup showed no evidence of factor V Leiden mutation or prothrombin gene mutation. Homocysteine and fibrinogen levels were in the normal range. There was no evidence of beta-2 glycoprotein, anticardiolipin antibodies, or lupus inhibitor promoting a hypercoagulable state. Antithrombin III and protein C levels were normal while protein S levels were reduced to 24%. Overall, normal protein S values range between 60% and 150%. With the diagnosis of dual sinus thrombosis, the patient was started on 5 mg of Eliquis (apixaban) to prevent future clot formation. Currently, she is adhering to her medication regimen while avoiding increased hypercoagulable states such as elective surgeries.

## Discussion

Along with having no current definitive cure, the intricacies of this rare qualitative blood disorder stem from the complications of the deficiency itself and its proposed prophylactic treatment plan. In protein S-deficient patients complicated with VTEs, the duration of therapy depends on severity. Patients with a VTE should initially be placed on anticoagulants such as warfarin with a heparin bridge to prevent skin necrosis, a severe side effect that can occur in this population [3]. Once the international normalized ratio (INR) is 2.0-3.0 for two days in a row, heparin can be discontinued and the patient is placed only on warfarin [6]. Severe VTEs found in cerebral or mesenteric veins require patients to be committed to a lifelong therapy of anticoagulants [6]. The main treatment for patients with protein S deficiency involves anticoagulation medications for the acute presentation along with prophylactic anticoagulation for life. This is the usual guideline for managing such patients [6]. Patients on any anticoagulation regimen must be monitored closely for severe unwanted bleeding. This condition can cause purpura fulminans in infancy, post-thrombotic phlebitis, and recurrent pulmonary embolism (PE), which can advance to cor pulmonale [7].

It's critical to educate patients on managing risk factors. The use of OCPs, hormone replacement therapy, and pregnancy creates a transiently hypercoagulable state [8], which works in synergy with protein S deficiency and can further increase the risk of VTEs.

The patient recalled having severe petechiae in the 1980s, which was mistakenly thought to be a factor VIII deficiency. Living in rural Alabama with limited access to healthcare hindered proactive screening methods and a thorough investigation. Despite having a family history of blood clots and presenting symptoms there was a lack of follow-ups after her brief incident. As fortunate as it sounds, the lack of VTEs before the age of 50 in this patient further contributed to the late discovery of her condition.

## Conclusions

Protein S deficiency is a rare disorder that tips the hematological balance in favor of hypercoagulation due to a lack of vitamin K-dependent plasma glycoprotein, also known as protein S. Acquired and congenital forms of the disease have similar complications such as post-thrombotic phlebitis and recurrent pulmonary embolisms. Congenital protein S deficiency has no definitive treatment but is managed with long-term anticoagulant therapy depending on severity, addressing risk factors that promote a hypercoagulable state, and encouraging routine follow-up in patients with a family history of blood clots. Identifying such rare disorders requires proactive screening and preventative medicine to be at the forefront of healthcare in underserved areas.
